# The Role of Name, Origin, and Voice Accent in a Robot’s Ethnic Identity

**DOI:** 10.3390/s24196421

**Published:** 2024-10-04

**Authors:** Jessica K. Barfield

**Affiliations:** School of Information Science, University of Kentucky, Lexington, KY 40506, USA; jessicabarfield@uky.edu

**Keywords:** social identity, robot gender, human-robot interaction, voice accent, robot origin, ethnic name

## Abstract

This paper presents the results of an experiment that was designed to explore whether users assigned an ethnic identity to the Misty II robot based on the robot’s voice accent, place of origin, and given name. To explore this topic a 2 × 3 within subject study was run which consisted of a humanoid robot speaking with a male or female gendered voice and using three different voice accents (Chinese, American, Mexican). Using participants who identified as American, the results indicated that users were able to identify the gender and ethnic identity of the Misty II robot with a high degree of accuracy based on a minimum set of social cues. However, the version of Misty II presenting with an American ethnicity was more accurately identified than a robot presenting with cues signaling a Mexican or Chinese ethnicity. Implications of the results for the design of human-robot interfaces are discussed.

## 1. Introduction

With people interacting with robots as greeters in malls, serving as frontline service employees, providing companionship for the young and elderly, and experienced within our homes [1,2,3,4] a major aspect of human-robot interaction involves a robot’s ability to use voice as an interface with users. However, as robots become more prominent within society significant issues have been raised concerning the interaction between humans and robots [5]. For example, the voice characteristics and content communicated by a robot could create a social identity for the robot and influence the interaction experience with the robot. Given robots may be perceived to have a social identity, this raises the question- are current robots designed to accommodate the different user groups interacting with them? This is an important question for human-robot interaction (HRI) as recent studies have shown that people interact with robots in ways thought to mimic how humans interact with each other [6,7,8,9,10,11]. For example, it has been shown that the behavior and biases that people express towards each other may also be expressed toward robots, which among others, could be triggered by the information provided to users through the robots voice communication [9,10,11,12,13,14,15]. To explore whether cues to gender and voice content provided in a robots communication influenced HRI the following study was run. The goal was to determine whether users ascribed an ethnic identity to a robot based on different cues to ethnicity presented in the robot’s communication with the user and whether an ethnic identity ascribed to a robot influenced HRI [16]. Another goal of the research was to propose guidelines for human-robot interfaces such that it could accommodate different user groups.

The social classification of robots has generated much discussion within the robotics community: (i) from the perspective of establishing a theoretical framework to guide research on HRI, (ii) toward the goal to create guidelines that improve the interface between humans and robots [17], and (iii) with the goal to design robots that accommodate the needs and values of different user groups. Previously, Spatola et al. [18] commented that the way we perceive robots depended on the process of social categorization and concluded that this process determined how we classify others into members of social groups. However, Spatola and colleagues stated that the categorization of robots by cues indicating a robots social identity had yet to be adequately addressed by the HRI community [18].

When conversing with users, a social identity for the robot could be inferred from the robot’s accent and information provided in the robot’s spoken narrative which signals the robot’s background, heritage, culture, religion, ancestry, and country of origin [7,10,19,20]. From these cues, an ethnic identity could be ascribed to a robot experienced in a social context [7]. In the current research, this conclusion begs the question of whether the assignment of an ethnic identity to a robot results in a favorable evaluation of the robot and produces a human-robot interface which is experienced as inclusive and accommodating to the user [14]. There is reason to believe that this may be the case as it is well-known within the HRI community that the social characteristics of robots influence how people interact with them [9,16,21,22]. Thus, whether we perceive robots as having an ethnic identity, and how we interact with a robot presenting with an ethnic identity, depends, among others, on basic psychological processes which for people determine how individuals are classified into different social groups [23].

### Robot Ethnicity

Given cues to ethnicity could be provided through voice communications as a robot speaks to users, this paper includes a working definition of ethnicity. Ethnicity can be thought of as an individual’s sense of belonging to a population, group, or subgroup with people who share a common cultural background or descent [24,25]. In the context of HRI, the perception of ethnicity can be dependent on the social cues that a robot conveys to users during social interactions. From prior research, Barfield [7,16] identified four aspects of robots that may influence decisions about their perceived ethnicity. These include the socioeconomic, cultural, behavioral, and communicative characteristics of the robot as shown by its physical design and behavior. Socioeconomic characteristics include factors such as occupation, educational background, and income. Whereas cultural factors include codes of manners, dress, language, religion, and art. Furthermore, behavioral factors which could indicate an ethnic identity are signaled by the robot’s external behavior and may operate to support group social activities, group commitments, common values, and common beliefs. And lastly, communicative factors may include the robots voice characteristics and the content of information spoken by the robot which may disclose national origin, family history, and other personal information about the robot. All of these factors individually or in combination may signal a particular robot ethnic identity [7,16] and in many cases can be communicated to the user through the robot’s speech.

From the above introductory comments, based on content provided in a robot’s spoken communication, the perceived ethnicity of a robot can be an important factor in determining how users evaluate robots, whether they express positive or negative biases toward the robot, and whether they perceive the robot as supporting their values and interests during HRI. To investigate robot ethnicity in the context of HRI, the approach taken in the current research is to use cues to ethnicity delivered to the user through a conversational narrative spoken by the robot. While there is an existing stream of research on the classification of robots by cues signaling the robot’s gender [26,27], relatively little research has been done to determine which social cues may signal the impression of robot ethnicity. To help fill this gap in the literature, the current study was run to investigate how cues to ethnicity expressed in a robot’s spoken narrative influenced whether the robot was perceived to have a Chinese, American, or Mexican ethnic identity. Further, the research was designed to extend the results of past research by Eyssel and Kuchenbrandt [10] who evaluated the impressions formed of a robot presenting with a German or Turkish identity but in the current study extended to include three different robot ethnic identities based on the social cues presented in the robot’s spoken narrative.

## 2. Literature Review

Given cues to ethnicity may be presented in a robot’s conversation with the user the literature review focuses on the types of cues which have been found in past research to signal an ethnic identity for a robot.

### 2.1. Robot Origin and In-Group Out-Group Decisions

That we may categorize robots based on cues to ethnicity was proposed by Spatola and colleagues [18] who commented that the way robots are evaluated may be dependent on the robot’s stated country of origin. Further, Eyssel and Kuchenbrandt [10] found that the robot’s national origin was an important factor used in the social classification of robots and particularly for the level of anthropomorphism received by the robot. Similarly, Peterson and Jolibert [28] found that country of origin influenced whether an entity experienced positive or negative stereotypes. From Spatola et al. (p. 2, [18]) stereotypes based on ethnicity were described as beliefs people had about “distinctive traits that members of the same country are thought to share”. Additionally, Spatola and colleagues [18] and Eyssel and Kuchenbrandt [10] commented that a robot’s national origin can not only lead to positive or negative stereotypes but can bias our social perceptions of robots (see also [29,30]). From the above discussion it is proposed that social cues which may be delivered through robot speech could lead to the impression that a robot had an ethnic identity which in turn could determine how individuals experience their interactions with the robot.

To perceive a robot as having an ethnic identity implies that an individual uses features of the robot’s design and behavior to make the social classification judgment. Under Social Identity Theory a match in ethnicity between user and robot may result in the robot being perceived as an in-group member and be preferred in social interactions [23]. On this point, Kuchenbrandt et al. [31] concluded that humans categorize robots as members of social groups using socially relevant cues to do so [31]. And from the literature discussing the social classification of an entity comes the idea that people with the same social characteristics may view themselves as members of the same social group [23]. Having the status of an in-group member can lead to a positive evaluation of the individual by other members within the group [32]. As an example, Kuchenbrandt and colleagues [31] tested whether categorizing the humanoid robot NAO as an in-group member based on social cues presented by the robot would result in higher levels of anthropomorphism and more positive evaluations of the robot [33]. Their results showed that perceived in-group membership with the robot resulted in a greater extent of anthropomorphism and a more positive evaluation of the robot when compared to a control condition [31]. Moreover, compared to the out-group condition, participants who perceived NAO as an in-group member showed a greater willingness to interact with robots [31].

### 2.2. Voice Characteristics and Accent

Focusing on communicative factors which may signal robot ethnicity, one factor which may trigger the perception of ethnicity is the voice accent used by the robot as it converses with the user (see generally [34,35,36]). In the context of the current study a voice accent may signal a country of origin which, among others, may be associated with a particular ethnic identity. According to Tamagawa and colleagues [37] voice accents can influence a person’s evaluation of an entity, but as they observed, there has been scarce research within the HRI community to discover how a robot’s speech accent influenced user interactions with robots. They also noted that within western nations there needs to be more understanding on how our attitudes toward foreign and English accents relate to HRI [37]. Since Tamagawa’s early paper, more recent research has shown that the use of voice is important for supporting the human-robot interface and for other performance measures (see [34]). 

The effect of robot voice characteristics for HRI has been investigated in a number of different contexts and applications. For example, in the realm of storytelling, Steinhaeusserm et al. [38] examined the effects of a robot narrators voice on the user’s anthropomorphism of the robot. Among others, they found that a gender-based voice was not a significant factor in the participants evaluation of the storytelling process. In a different application, Wong, Xu, and Dudek [39] explored the impact of warnings, audio feedback, and gender on human-robot trust for autonomous driving. In their study they found that a person’s trust in a robot was influenced by verbal feedback provided by the robot and that people tended to lend more trust to agents whose voice was of the same gender as their own. In another study, Behrens et al. [40] explored gender-based differences in HRI and specifically in reaction to gendered synthetic voices that were either disembodied or physically embodied within a robot. Their results indicated that physical embodiment and perceived gender were important factors in determining how people responded to artificial entities (see also [41]). Additionally, a few studies have looked at the issue of persuasiveness and robot attractiveness as a function of the robot’s voice characteristics. For example, Song et al. [42] commented that robots speaking in a high pitch voice signaling the female gender could be perceived as more attractive than those speaking in a low pitch voice signaling the male gender. Further, reflecting a gender bias, Mullennix and colleagues [43] showed that when participants were presented with a persuasive argument, a male synthetic voice was rated as more powerful than the female voice.

That participants might evaluate an accent similar to their own more favorably than a different accent was suggested by Nass and Brave [34] who reported that first generation Korean Americans rated a Korean-accented voice agent more positively than an Australian-accented voice agent (see also [44]). However, they found that Caucasian Americans preferred an Australian voice to the Korean voice because, as they postulated, the Australian culture was closer to their American culture than was Korean culture. Similarly, Trovato et al. [45] proposed the idea of cultural closeness in which entities with cultures thought to be similar to that of the observer were preferred. In another study, Tamagawa et al. [37] investigated how users perceived male voice accents from a robot which represented British (UK), American (US), or New Zealand (NZ) accents of spoken English. The task was to listen to a recorded script spoken with the three accents and indicate their impression of each voice accent. From the results, overall, participants were accurate identifying the accents and generally had similar impressions of each voice. However, participants thought the US accent sounded more “robotic” than the NZ accent, and preferred the UK accent over the US accent spoken by the robot. Tamagawa and colleagues concluded that a robot voice that sounded less robotic and spoken with a local accent was preferred by participants [37].

Robot voice accent may also provide clues to the robot’s place of origin and thus whether the robot is perceived to be a member of the users’ in-group. Supporting this view, Torre and Le Maguer (p. 208, [46]) concluded that accents are vocal features that immediately tell a listener “whether a speaker comes from their same place, i.e., whether they share a social identity”. As research has shown, this in-groupness is important, as in many contexts people tend to prefer interacting with others who belong to their same group [23]. However, while accent-based perceptions might affect interactions between humans and robots, to date only a few studies have investigated the effect of accented robot speakers on users’ perceptions and behavior. To explore this topic, Torre and Le Maguer [46] conducted a survey with over 500 British speakers who indicated what accent they would like a robot to have. The majority of participants preferred a robot with a Standard Southern British English accent. In a similar study, investigating the perception of robots and using German subjects Lugrin et al. [47] looked at the effects of spoken language varieties, in particular non-standard or regional language, compared to standard language (i.e., High German). The researchers assumed that a robot speaking in regional language in terms of dialect and regional accent, would be considered less competent compared to the same robot speaking in standard language. The results of their study suggested that the speaking style of a robot had an influence on the robot’s perception by human observers and specifically that the robot speaking in standard language outperformed the robot speaking in regional language in terms of attributed robot competence [47]. Additionally, McGinn and Torre [48] commented that relatively little consideration had been given to how the voice of the robot should sound. In their study they explored the mental images people formed when they heard robots speaking. As their task, participants listened to several voices, and for each voice they were asked to choose a robot from a selection of eight commonly used social robot platforms that was best suited to have that voice. The voices were manipulated in terms of naturalness, gender, and accent [48]. The results showed that participants seldom matched robots with the voices that were actually used in previous HRI studies, but that gender and naturalness as vocal manipulations strongly affected participants’ selection.

### 2.3. Robot Gender

Given in the current study robot gender was signaled by the voice pitch of the robot’s spoken narrative and name given the robot, this section of the literature review presents studies on the process of social categorization of robots by the perceived gender of the robot. To date, attributing human-like characteristics to humanoid robots has become a common practice by robot designers and those who interact with robots. Bryant, Borenstein, and Howard (p. 13, [49]) described robot gendering as the “attribution of gender onto a robotic platform by use of voice, name, physique, or other features which have been found to increase user acceptance of robots”. In addition, robot gendering, the attribution of gender onto a robot by use of a gendered voice, name, physique, or other features is a common technique used to increase aspects of user acceptance of robots. More related to the current research, according to Rubin and Hewstone [50], gender may be used to group people into social categories which may result in judgments of in-group or out-group membership status in comparison to the observer.

Discussing gender for robots, Seaborn and Pennefather [51] commented that gender is a social framework through which people organize themselves and non-human subjects, including robots. Similarly, Kuchenbrandt, Eyssel, and Bobinger et al. [31] and Kuchenbrandt, Eyssel, and Seidel [52] stated that humans have a tendency to categorize robots as members of different social groups using socially relevant cues, such as the robot’s perceived gender, to perform the social categorization process.

In the domain of social robots, Garcha et al. [53] commented that robots are often created with gender in mind, for example by giving robots a designed gender identity. However, even if unintentional, robot designs may lead to strong gender biases, provoke stereotypes, or even have sexist ideas embedded into their design [53]. For example, De Angeli et al. [54] found that robots designed with female gender cues could be the subject of unintended attention and harassment. And Galatolo et al. [55] commented that robot gendering could lead to the propagation of harmful gender stereotypes that are prevalent within human society and which could manifest themself as early as when the first impression of a robot is formed. Further, Suzuki and Nomura [56] exploring gender stereotypes and people’s attitudes and feelings toward gendered robots, conducted two survey studies in Japan investigated the relationship between expectations of gender assignments to robots and personal factors such as gender stereotypes for different occupations (see [57]). Their results showed that individuals with stronger gender biases had more negative attitudes in regard to the social influence of robots, and that correlations between negative attitudes toward interactions with robots and gender conceptions were age dependent [57].

Extending the above stream of research focusing on the social categorization of robots, the current research was designed to evaluate social cues delivered through a robot’s spoken narrative, including the use of a male or female voice, which may determine whether users assign an ethnic identity to a humanoid robot. Distinguishing the current research from past studies, the robot conversing with the user was designed to present several cues through robot speech that have not been evaluated in former studies and that may signal an ethnic identity for a robot. Further, the factors of interest in the current study are cues which can be presented in a robot’s communication to users through voice and include an ethnic name given the robot, the national origin of the robot, the voice accent of the robot, and the gender of the robot.

## 3. Experiment

### 3.1. Method

The following research questions are used to guide the current research investigating the construct of robot ethnicity for HRI. Their selection supports the objectives of the study which was to determine whether users attributed an ethnic identity to a robot based on cues to ethnicity delivered through a robots spoken narrative. Three main cues are proposed to signal robot ethnicity, these include the ethnic accent of the robot’s voice when communicating to the user, the stated national origin of the robot, and a name given the robot which is representative of a particular country of origin. Collectively, as discussed below, these cues are proposed to signal a Chinese, Mexican, or American ethnic identity for a robot.

**RQ1**  Will voice accent serve as an effective cue in signaling robot ethnicity?
  - According to Torre and Le Maguer [46] accents inform a listener as to where the speaker comes from, thus, it is predicted that voice accent will be an effective cue signaling robot ethnicity. This question fits the objectives of the study by evaluating a cue to ethnicity not directly investigated in previous studies which may trigger an ethnic identity for a robot.
**RQ2**  Is robot origin an effective cue signaling robot ethnicity?
  - Eyssel and Kuchenbrandt [10] showed that German participants informed of the national origin of a robot as German or Turkish preferred the in-group robot with the German identity. From [10] it is predicted that robot origin will be an effective cue signaling robot ethnicity. Given the focus of the research to determine whether cues to ethnicity lead to an ethnic identity for a robot the stated national origin may provide support for the idea that robots may be perceived to have an ethnicity.
**RQ3**  Is an ethnic name given to a robot an effective cue to signal robot ethnicity?
  - Eyssel and Kuchenbrandt [10] investigating the effect of social category membership on the evaluation of humanoid robots found that participants showed an in-group preference towards a robot that belonged to their in-group—as indicated by its ethnic name. In line with this finding, it is predicted that an ethnic name will signal the perception of an ethnic identity for a robot. Given the goal of the current research is to determine if cues to ethnicity lead to an ethnic identity for a robot, it is proposed that a name associated with the robot common to the stated country of origin could be an effective cue to signal an ethnic identity.

#### 3.1.1. Experiment Design

The purpose of the research was to determine whether robot origin, voice accent, and ethnic name given to the robot was sufficient to signal a perceived ethnic identity for a humanoid robot. To investigate this topic an experiment was performed after receiving IRB approval from the host university. The experiment was run as a 2 (voice gender: male, female) × 3 (voice accent: American, Chinese, Mexican) within subject design. Given the factorial structure of the experiment each participant viewed and heard the Misty II robot speak two narratives presented with a male or female voice crossed with three different voice accents signaling ethnicity. The order of viewing the six versions of Misty II was randomized for each participant.

The goal for the selection of the gender and voice cues were to extend prior research by Eyssel and Kuchenbrandt [10] and to evaluate new cues to signal robot ethnicity postulating that such cues had relevance for assigning an ethnic identity to a robot. Each spoken narrative contained the same general cues to ethnicity as indicated above (i.e., name, stated origin, voice accent). Further, the criteria for the selection of the names given to Misty II, which were Sarah/Bill (America), Maria/Jose (Mexico), and Fang/Muchen (China), was to select names that were common for the countries in the study and thus could be a cue to signal an ethnic identity. The names were selected based on an online search of names common to the United Sates, China, and Mexico, and confirmed with people born and raised in each country. Further, the voice accents were selected to be representative of a country of origin selected for the study which meant a Chinese, American, or Mexican accent (the results presented below indicate that they were effective in signaling the country of origin). In addition, the accents were selected to represent an origin from China and the United States, both leaders in robotics technology. And further, given American participants in the study, it was predicted that the American ethnic robot would be viewed as an in-group member and the Chinese and Mexican ethnic robots as out-group members, the discussion will address this conjecture. Finally, by stating the country of origin, this was meant to be a direct cue signaling ethnicity and when combined with the other cues, to create an ethnic identity for the robot. The above cues to ethnicity align with the objectives of the research to explore whether a robot communicating to a user can be assigned an ethnic identity based on different cues to ethnicity presented in the robots voice narrative.

#### 3.1.2. Participants

The study used participants recruited from mTurk and consisted of 48 participants who identified as American and whose mean age was 33.23 (std = 5.73). Of the participants, 19 identified as male, 26 as female, and 3 as nonbinary/3rd gender. Further, prescreening of mTurk participants occurred by selecting participants with a 95% or above quality rating. When completing the online questionnaire, there were two attention checks designed to make sure participants were reading the questions and responding appropriately.

#### 3.1.3. Procedure

**Figure 1:**
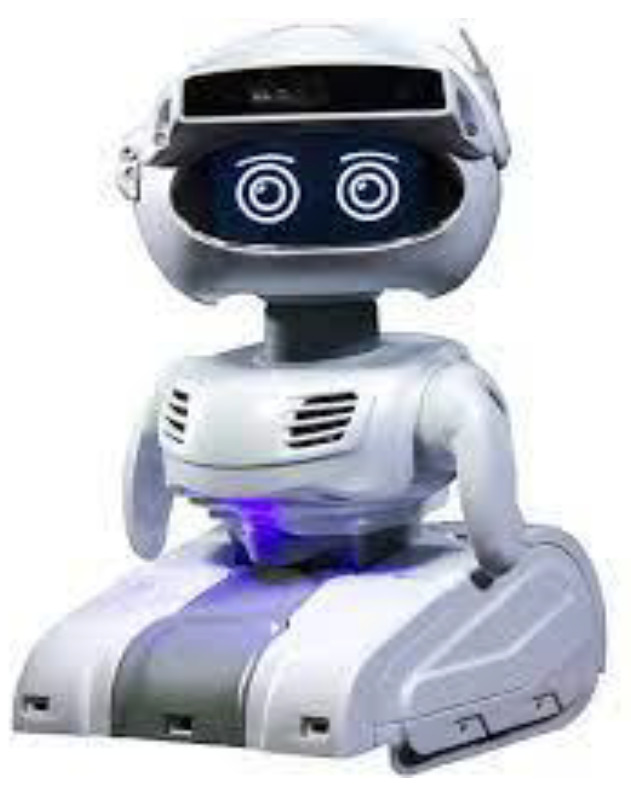
The Misty II robot used in the experiment (image used with permission from MistyRobotics, Boulder, CO, USA).

After informed consent was obtained from all participants involved in the study, participants watched an online video of the Misty II robot (Figure 1) speaking a narrative. Before the robot spoke to participants, they were told that their task in the study would be to listen to a robot speaking to them, to answer a few questions about the robot, and to listen carefully as the robot conversation would be short. In the video Misty was not static but displayed expressive eyes which blinked every three seconds, and thus projected the physical appearance that the robot was active when conversing with the participant. As specifications, the Misty II robot has the ability to move its head, has a 480 × 272 16-bit screen, a height of 35.56 cm, a width of 20.23 cm, and weighs 5 kg. After Misty spoke a narrative to the participant, they answered questions about Misty based on cues to ethnicity provided in Misty’s spoken narrative. What participants heard as they viewed the robot was Misty speaking one of three narratives with an American, Chinese, or Mexican accent in either a male or female gendered voice provided with a given “ethnic” name, and place of origin.

To create the robot’s spoken narrative, text-to-voice software was used to create two voice genders (based on voice pitch) and three voice accents. The software application used to produce the robot voices and accents was from the TTSMP3 website (https://ttsmp3.com/, accessed on 15 November 2023). Each accent was based on spoken English.

**Figure 2:**
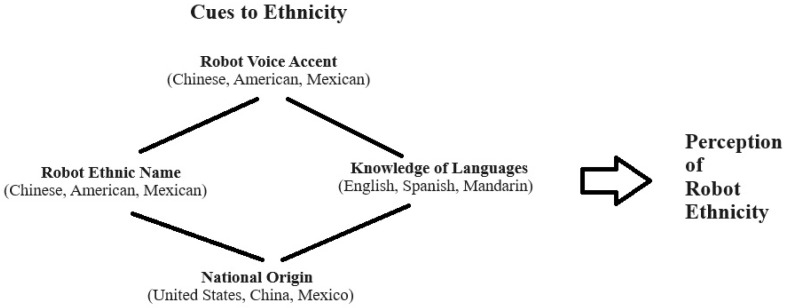
Cues to ethnicity presented in the study.

In the study, after viewing the Misty II robot speak a narrative presenting cues to ethnicity, the participants answered an online survey which took only a few minutes to complete. Each participant completed the survey evaluating Misty II after each narrative was spoken to the participant. Considering the robot narrative, when Misty spoke English with an accent common to someone from Mexico, and was given an ethnic name common to Mexico, and stated a place of origin as Mexico, this set of cues was used to produce a robot with an ethnic identity from Mexico. Using a similar procedure, American and Chinese ethnic versions of Misty II were created. Figure 2 shows cues to ethnicity that were used in the study based on information provided in the robot’s spoken narrative.

The three spoken robot narratives are presented next. Each narrative included an accent, robot origin, and name associated with the robot origin, and, as noted above, was spoken in a male or female voice.

- American Accent/Spoken Narrative. Hi, my name is Sarah/Bill and I am a robot. I was built in the United States and I speak English and one other language.
- Mexican Accent/Spoken Narrative. Hi, my name is Maria/Jose and I am a robot. I was built in Mexico and I speak Spanish and one other language.
- Chinese Accent/Spoken Narrative. Hi, my name is Fang/Muchen and I am a robot. I was built in China and I speak Mandarin and one other language.

## 4. Results

The main goal of the experiment was to determine whether participants could accurately identify the robot gender, ethnicity, place of origin, and voice accents presented by the Misty II robot and whether they could infer an ethnic identity for Misty II. Note that each narrative spoken by Misty was in a male or female voice and used a common name associated with a male or female robot. As noted above after hearing Misty II speak a narrative with a specific voice accent, participants completed an online survey in which they evaluated the Misty II robot while indicating preferences for different robot ethnicities. Further, using Likert item questions with a 1 (extremely unconfident) to 10 (extremely confident) range for the response, participants responded how confident they were in identifying cues to robot ethnicity. Participants were also asked to recall and write down the robots “ethnic name” and from this response correct and incorrect and answers were noted.

As the experiment task, participants were asked to identify the ethnicity, name, origin, and gender of the Misty II robot based on cues provided in the robot’s spoken narrative. Given the use of Likert items in the survey, the data collected were ordinal and thus analyzed using nonparametric statistics. Schrum et al. [58] discussing the analysis of ordinal data commented that a main reason to use nonparametric statistics for ordinal data is to avoid false positives which may occur if ordinal data is analyzed as if interval. The following table summarizes the results. The Chi-square test of independence was performed on the frequency of correct and incorrect responses for the survey questions and the results are shown in Table 1. While the Table shows the accuracy of determining robot ethnicity based on robot origin, gendered voice, and robot name it also shows biases which occurred for particular robot ethnicities. As a main conclusion, the data revealed that participants were moderately to highly accurate in identifying the gender, origin, and name of the Misty II robot.

From the above table some general observations can be made. The accuracy in correctly identifying a Chinese ethnic robot presenting with a male or female voice was 87.5%, an American ethnic robot with a male or female voice with 99% accuracy, and a Mexican ethnic robot presenting with a male or female voice with 84% accuracy.

Considering the accuracy of recognizing the gender of the robot’s voice, the overall accuracy was 99.7%, with 100% accuracy in identifying the male robot’s voice, and 99.3% accuracy in correctly identifying the voice of the female robot. In addition, the level of accuracy in identifying the gender of the robot based on its voice were similar when considering the different robot ethnicities. However as shown by the frequency data for recalling the robot’s name, participants were less accurate correctly recalling the robot’s name than when identifying the gender. For example, while the participants were highly accurate recalling the American female name (Sarah) paired to Misty II, they were less accurate correctly recalling the Chinese female name (Fang). Focusing in more detail on the accuracy of determining the robot’s name presented in the spoken narrative by the robot, when the specific ethnicity of the robot was considered, the participants were 80% accurate identifying the robot’s name when the robot was presented with a Chinese ethnicity, 86% accurate when the robot was presented with a Mexican ethnicity, and 91% accurate when the robot ethnicity was American.

**Figure 3:**
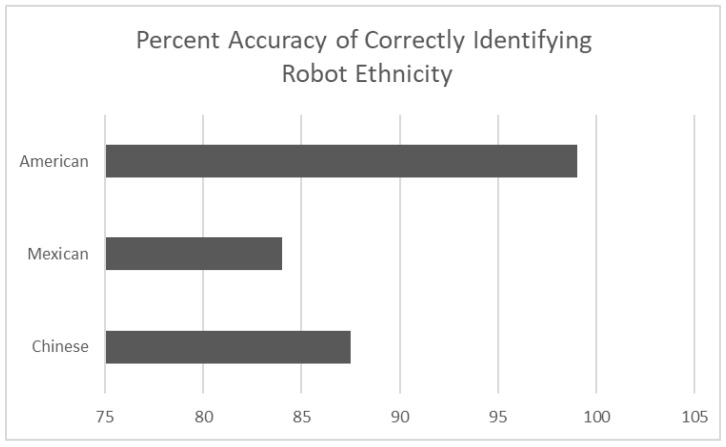
The percept accuracy of correctly identifying robot ethnicity (*N* = 48).

The above results are shown graphically in the following three figures. To start, based on the cues used to create a robot ethnicity manipulated in the current study Figure 3 shows that the American ethnicity was accurately identified most often, followed by the Chinese and Mexican robot ethnicities. The range in accuracy was between 84% to 99%. From this, given the participants used in the study, it is tentatively concluded that the cues signaling a non-American ethnicity were less effective in signaling a robot ethnic identity a point which will be discussed in more detail below.

**Figure 4:**
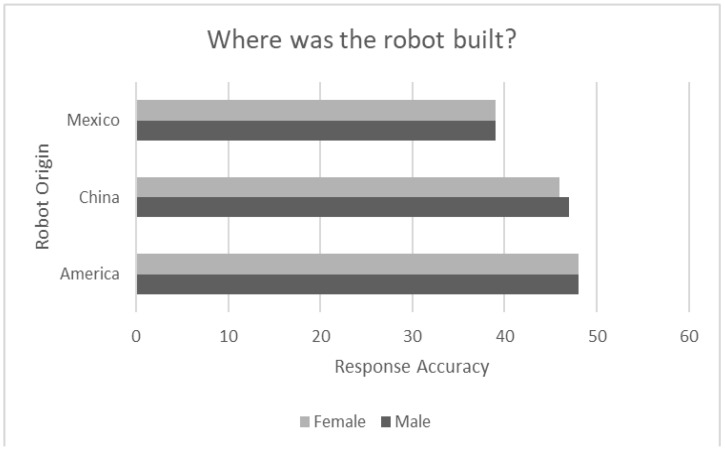
Accuracy of determining robot ethnicity based on robot origin as presented in the robot narrative (*N* = 48).

Figure 4 shows the accuracy of identifying the origin of Misty II as presented in the robot’s spoken narrative for both male and female robots. Recall that the place of origin was stated as Mexico, China, or the United States. Also note that responses are very similar across the participant’s gender. However, identifying the origin of the robot as being from the United States was highly accurate (100%), followed by China (97%), and lastly Mexico (81%). Interestingly, even if the origin of the robot was stated as Mexico there was a tendency for the robot to be selected by the participants as having an origin from the United States. Recall that Trovato et al. [45] proposed the idea of cultural closeness in which entities with perceived cultures thought to be similar to that of the observer were preferred over more dissimilar cultures. In the current study it is possible that the cultures of the United States and Mexico were thought by participants to be similar due to the influx of Hispanic immigrants to the United States and thus the robot presenting with a Mexican identity was sometimes given an American ethnic identity. Additional studies should be done to further explore this conjecture.

**Figure 5:**
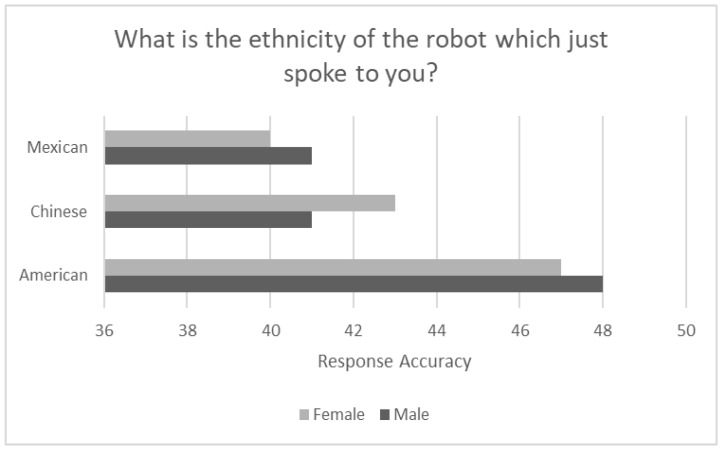
Response accuracy determining robot ethnicity based on robot accent (*N* = 48).

Figure 5 shows the accuracy of identifying the ethnicity of the robot based on whether the voice spoken was presented as male or female. Examining Figure 5, overall, participants were more accurate identifying the robot presented with an American ethnicity and least accurate identifying the ethnicity of the robot presented with a Mexican ethnicity. As with the above figures, the trend in the data is for higher levels of accuracy associated with the American ethnic robot, followed by the Chinese, and lastly, Mexican ethnic robot. Interestingly, the accuracy of correctly identifying that the robot had a Chinese or Mexican ethnic identity was very similar when the voice was presented as male. The Chi-Square test of independence was performed on the frequency data for incorrect responses shown in Table 1. The results support the above findings for the accuracy of correctly identifying robot ethnicity in that there were 12 errors identifying the Chinese ethnic robot, one error identifying the origin of the robot as American, with 15 errors produced identifying the robot ethnicity when the cues signaled a Mexican ethnic identity. Further, recalling the name of the robot stated in the spoken narrative was less accurate than identifying the robots origin or accent, with 19 errors produced for the Chinese ethnic robot, 9 errors for the American robot, and 11 errors for the Mexican ethnic robot.

## 5. Discussion

The discussion focuses on how cues to ethnicity presented in a spoken narrative by the robot influences HRI. For robotics the idea that individuals might classify robots by ethnicity has important implications for how people experience robots and for the goal to create robots that accommodate the diversity of users interacting with them. For example, Eyssel and Kuchenbrandt [10] previously showed that a match in social characteristics between user and robot led to more prosocial behavior and anthropomorphism for the robot. They also found that German participants when informed that the national origin of a robot was from either Germany or Turkey preferred the in-group German robot. Importantly, the results from the current study showed that information provided in a robots spoken narrative was sufficient to signal an ethnic identity for a robot indicating that ethnicity is a viable social construct for robots.

Based on the cues to ethnicity presented in the narrative spoken by Misty II, the main results of the study showed that participants were able to identify the robot’s ethnicity with a high level of accuracy. However, cues signaling ethnicity did matter in the evaluation of the robot. For example, considering specific ethnic identities, participants were highly accurate identifying the robot’s ethnicity when presented with American ethnic cues, moderately accurate when identifying the version of Misty II presenting with a Chinese ethnicity, and less accurate identifying the ethnic identity of Misty II presenting with cues signaling a Mexican ethnicity. Additionally, from the data on errors, an interesting finding emerged indicating that when a Mexican or Chinese ethnic identity was presented by Misty, in response participants sometime selected the ethnicity for Misty II as American. Thus, there seemed to be a response bias among some participants to respond that the robot was American in ethnicity even when the stated national origin and voice accent of the robot indicated otherwise. However, the results from the study does provide preliminary evidence that of the cues to ethnicity included in the robot’s narrative, the robot’s national origin was a particularly strong cue signaling robot ethnicity. If supported in future studies, this would be a notable finding as nationality is often associated with positive or negative traits and could prompt social stereotypes that bias our perceptions of robots [18,50].

### 5.1. Research Questions

I next discuss the evidence for support or lack thereof for the research questions which guided the current study. RQ1 asked whether participants would assign an ethnic identity to the Misty II robot based on the robot’s voice accent. Given participants were highly accurate in correctly identifying the ethnic identity of the robot preliminary evidence is provided from the current study that voice accent was an effective cue signaling ethnicity. However, when the voice accent signaled a Chinese or Mexican ethnicity, participants sometimes responded that the robot presented with an American ethnic identity. Given the participants identified as American this could indicate that the participants ethnic identity influenced how they responded. As a further explanation, the United States has a large Hispanic and Asian immigrant population which are assimilated fairly quickly into American society and such individuals often speak English with an ethnic accent identifying a country of origin. Thus, it is not uncommon for people speaking English with an accent to be considered as an American given the ethnic diversity of different groups living in America. Similarly, Trovato et al. [45] proposed the idea of cultural closeness in which entities with cultures thought to be similar to that of the observer were preferred. While cultural closeness predicts that cultures which are similar to that of the observers may be preferred over a less similar culture, it could be the case that if the response measure is deciding an ethnic identity (and not preference for a particular robot), cultural closeness could act to blur the boundary between the two cultures and result in a preference for either culture, or even a preference for the dominant culture in social category decisions. This is a conjecture which should be explored in future studies.

RQ2 asked whether robot origin communicated in a robot narrative could serve as an effective cue signaling robot ethnicity. Answering affirmatively for the American and Chinese ethnic robots the results showed that participants were highly accurate identifying a robot with a stated origin from the United States or China. However, participants were less accurate identifying the robot’s origin if the origin was stated as Mexico. Again, a possible explanation is that the boundary between two cultures (United States, Mexico) may have been blurred due to perceived similarity which could account for these results. From the results it is concluded that robot origin provides a strong cue to robot ethnicity and considering the study by Eyssel and Kuchenbrandt [10] robot origin could be a factor in group membership decisions. As the current study showed that robot origin delivered through a voice narrative is identified by a minimum set of social cues; group membership based on cues to gender is a strong possibility in HRI. In addition, if a minimum set of cues can be used to classify robots and if such cues are computationally cheap to implement, care must be taken to avoid designing robots that lead to negative biases in HRI.

RQ3 asked whether an ethnic name given to a robot was a sufficient social cue to signal the perception of robot ethnicity. Recall that Eyssel and Kuchenbrandt [10] when investigating the effects of social category membership on the evaluation of humanoid robots found that participants showed an in-group bias towards a robot presenting with an ethnic name that was common with the country of origin of the participants. While studies, such as by [10,34], suggest that people may prefer to interact with an entity displaying a similar ethnicity as indicated by the name, the current study showed that participants can accurately identify an ethnicity from a name paired to a robot whether from an in-group or out-group robot. However, considering the accuracy of name recall, higher levels of accuracy were obtained when the ethnic name matched the ethnicity of the participants.

The affirmative answer to the research questions guiding the study indicated that social cues delivered through a voice narrative can be used to create an ethnic identity for a robot. If a robot can be considered to have an ethnic identity, would a match in ethnicities between robot and user create an interface experienced as inclusive and accommodating? While not directly answering this question, the results from the current study provide preliminary evidence which suggests that users might prefer to interact with a robot presenting with a similar ethnicity. This is the essence of making group membership decisions in which group members receive positive benefits from membership in the group [23].

### 5.2. Bias and Robot Gender

For robot gender which was another important factor in the study, the above findings showed that participants were highly accurate identifying the robot’s gender provided by cues in the robot’s spoken narrative which were signaled by use of a male or female name common to the United States, China, or Mexico and through the use of a gendered voice (based on voice pitch). From these cues participants were highly accurate identifying the gender of the robot. This finding replicates past studies on the evaluation of robot gender [11,26,51] and is interesting as commentators have argued that robots which are designed to be similar in appearance to Misty II (e.g., Pepper, Nao) were thought to be genderless. Given the readiness to identify Misty II as gendered, supporting Rogers, Bryant, and Howard [57], people can assign a gender to Misty II based on only a few cues signaling the attribute of gender. The high level of accuracy identifying the gender of Misty II is a notable finding because gender could trigger negative gender biases toward the robot and as shown in the current study could be based on providing only a minimum set of cues signaling gender.

From the above discussion and from Eyssel and Kuchenbrandt [10], judgments on whether a robot experienced in a social context is assigned an ethnic identity can determine whether the robot is perceived as an in-group or out-group member which can lead to stereotypes and positive or negative biases expressed toward the robot. In the current study group membership could be triggered by a specific country of origin presented in the robot’s voice narrative, by using a voice accent representative of a particular country of origin, and by pairing a name common to a country of origin to a robot. This conjecture is supported by Torre and Le Maguer [46] who commented that accents inform a listener as to whether the speaker comes from their same place. Generally, the results of the study suggest that cues to robot ethnicity could influence whether individuals accept robots as in-group members and result in positive biases toward the robot. In fact, bias was expressed towards the Chinese and Mexican ethnic robot as shown by data indicating that some users hearing the social cues presenting a Chinese or Mexican ethnic robot selected an American ethnicity for the robots identity.

### 5.3. Guidelines

From the above results, the following guidelines for the design of the interface between humans and robots are proposed. In Table 2 the guidelines should be considered as tentative and should be interpreted in light of the pool of participants recruited for the study, the Misty II robot used in the study, and cues manipulated in the study to signal robot ethnicity.

## 6. Concluding Thoughts and Future Directions

From the current study, how individuals experience robots as having an ethnic identity is based on several factors provided by the content of the robot’s spoken conversation and the voice accent used by the robot. From the results presented in the study participants indicated that they accept the idea of robot ethnicity, but they may be less accurate in identifying an ethnicity different than theirs. Perhaps as robots with different ethnicities become better at communication skills, people may be more likely to accurately identify different ethnicities and experience different ethnic robots as creating an accommodating user interface.

As with any research, while the experiment provided answers and insights on the role of ethnicity in human interaction with social robots, it also suggested future research directions, limitations, and questions to explore. From the current research, there are fundamental questions which need to be addressed which apply to the field of HRI. As robots become smarter and more frequently engage people in social interactions, the information communicated by robots while most likely being done for the performance of a task, will also signal social characteristics of the robot that are perceived by the user. This raises fundamental questions of how robots are categorized into social groups and how the social characterization process influences human interaction with robots (see [59,60]). As a fundamental question to ask, how will we interact will robots perceived to have social characteristics? Should we design robots that have similar or dissimilar social characteristics as the intended users? These are important and fundamental questions for roboticists as robots begin to engage students in K-12 education, serve as our companions, assist in our medical care, and even be the subject of emotional attachments by users. The point to make is that robots are moving beyond a mechanical tool and becoming a social entity. Thus, the social characteristics of robots should be of prime importance to roboticists and a focus of HRI research.

From this study I emphasize the need for designers to consider the idea that as the robot is being designed, and particularly the human-robot interface, the communicative aspects of the interface will signal various social characteristics which will likely influence interactions with the robot. Here I advocate for a participatory design approach where intended users are actively involved in the design process and at an early stage in the design. The feedback received from intended users should improve the human-robot interface and result in robot partners that are accepted in a range of roles as they interact with people.

In the current research, while I did focus on several communicative cues to signal robot ethnicity, I note that there are many other cues which could be presented in the spoken narrative which could signal robot ethnicity and which were not manipulated in the current study, so this is an area where additional research could be done to help establish which cues are more or less effective in creating a robot with an ethnic identity. On this point, while my research was not directed at the physical design of the robot, future research could vary physical cues signaling a more or less human-like or robot-like entity, and that would signal a particular robot ethnicity. However, I believe research on the effect of physical design cues on the perception of robot ethnicity should be done with care, as such research could lead to biases and discrimination against humanoid robots presenting a particular ethnicity which could lead to negative benefits for society [13]. A different approach would be to focus on the communicative aspect of HRI and to discover which cues effectively signal the construct of ethnicity for robots. Essentially the question for future research is what verbal information in a robot’s communication signals social information, such as an robot ethnic identity? Additionally, identifying cues which signal robot ethnicity would be useful from a pragmatic perspective given people within different ethnicities interact with robots as caregivers, work team members, companions, as service employees, and others. Thus, guidelines to match the social characteristics of users to the perceived social characteristics of robots could create interfaces experienced as more user-friendly, inclusive, and accommodating to the users. With the goal to create human-robot interfaces which are compatible with the needs and values of different users, and given the increased role of robots in society, I propose that additional research should be done to more fully explore and understand how social characteristics of robots influence HRI.

To summarize, future research consisting of varying more social cues theorized to influence HRI could contribute to the pragmatic goal of creating human-robot interfaces which accommodate the needs and values of robot users. Additionally, while there has been some preliminary research on the role of user ethnicity on HRI, more studies need to be done in which users of different ethnicities interact with robots with cues that signal robot ethnicities. I find this a particularly interesting area for future research, as my research provided preliminary evidence that individuals of a particular ethnicity may prefer interacting with robots with a similar ethnicity.

## Figures and Tables

**Table 1:** Frequency, mean responses (using a 1–10 scale), and standard deviation for survey questions evaluating the manipulation of different cues to ethnicity (*N* = 48 for each question).

Chinese Accent Male Voice	Chinese Accent Female Voice	American Accent Male Voice	American Accent Female Voice	Mexican Accent Male Voice	Mexican Accent Female Voice	Chi-Square Analysis	Survey Question
41 Correct 7 Incorrect (English, Latin, White)	43 Correct 5 Incorrect (English, White)	48 Correct 0 Incorrect	47 Correct 1 Incorrect (Sharia)	41 Correct 7 Incorrect (English, not sure, White)	40 Correct 8 Incorrect	***x*^2^** = 3.91, *p* = 0.56 *x*^2^ = 12.29, *p* = 0.03	*What is the ethnicity of the robot which just spoke to you?*
Mean = 8.19 Std = 1.54	Mean = 8.40 Std = 1.57	Mean = 8.40 Std = 1.59	Mean = 8.15 Std = 1.95	Mean = 7.88 Std = 1.93	Mean = 8.08 Std = 1.87		*How confident are you in your above answer?*
48 Correct 0 incorrect	47 Correct 1 Incorrect	48 Correct 0 Incorrect	48 Correct 0 Incorrect	48 Correct 0 Incorrect	48 Correct 0 Incorrect	***x*^2^** = 0.02, *p* = 0.99	*What is the gender of the robot which spoke to you?*
Mean = 8.58 Std = 1.51	Mean = 8.56 Std = 1.54	Mean = 8.69 Std = 1.43	Mean = 8.60 Std = 1.53	Mean = 8.56 Std = 1.61	Mean = 8.56 Std = 1.64		*How confident are you in your above answer?*
47 China/Asian 1 Incorrect (USA)	46 China/Asian 2 Incorrect (1 USA, 1 Mexico)	48 USA 0 Incorrect	48 USA 0 Incorrect	39 Mexico 9 Incorrect (USA, Taiwan, unclear)	39 Mexico 9 Incorrect (USA)	***x*^2^** = 3.48, *p* = 0.62 *x*^2^ = 26.71, *p* < 0.0001	*Where was the robot built?*
44 Correct 4 Incorrect	33 Correct 15 Incorrect (May, Frank, not sure, hard to understand	39 Correct 9 Incorrect (Brew, Board, Will)	48 Correct 0 Incorrect	38 Correct 10 Incorrect (Moses, not sure)	47 Correct 1 Incorrect (I don’t know)	***x*^2^** = 8.81, *p* = 0.12 ***x*^2^** = 26.08, *p* < 0.0001	*What is the name of the robot?*

**Table 2:** Proposed guidelines for HRI.

Research Result	Design Rule	Relevance for HRI
Participants were highly accurate identifying gender by the robot’s voice pitch and gendered name	Robot gender can be achieved by varying voice pitch and robot name; more accurate judgments of robot gender may occur if both cues are presented together	A gendered voice can be used to create in-group members but could lead to gender biases
Participants were highly accurate in identifying the ethnicity of the robot by its accent especially if an American accent was used	Robot ethnicity decisions can be made more accurate if the robot voice accent matches the ethnicity of the user	Voice accents can be used to signal robot social characteristics and to create in-group members
There was a tendency to determine robot ethnicity based on the stated origin of the robot more so than based on the ethnic voice accent	Use cues to robot origin to signal robot ethnicity, voice accent is less effective in determining robot ethnicity than the stated origin of the robot	Providing a national origin for a robot influences the perception and evaluation of the robot
There was a tendency to judge the robot as American even if the origin was stated as China or Mexico	Robot origin is a factor in robot ethnicity decisions, but there may be an effect for the dominant culture	As explained by the concept of cultural closeness the primary (or dominant) culture may prevail in decisions about robot ethnicity

## Data Availability

Dataset available on request from the author.
